# Gene Expression Profiling via Multigene Concatemers

**DOI:** 10.1371/journal.pone.0015711

**Published:** 2011-01-18

**Authors:** Kai Jin, Xiaoli Zheng, Yuxian Xia

**Affiliations:** 1 Genetic Engineering Research Center, School of Bioengineering, Chongqing University, Chongqing, People's Republic of China; 2 Chongqing Engineering Research Center for Fungal Insecticide, Chongqing, People's Republic of China; 3 Key Laboratory of Gene Function and Regulation Technologies under Chongqing Municipal Education Commission, Chongqing, People's Republic of China; University Hospital Vall d'Hebron, Spain

## Abstract

We established a novel method, Gene Expression Profiling via Multigene Concatemers (MgC-GEP), to study multigene expression patterns simultaneously. This method consists of the following steps: (1) cDNA was obtained using specific reverse primers containing an adaptor. (2) During the initial 1–3 cycles of polymerase chain reaction (PCR), the products containing universal adaptors with digestion sites at both termini were amplified using specific forward and reverse primers containing the adaptors. (3) In the subsequent 4–28 cycles, the universal adaptors were used as primers to yield products. (4) The products were digested and ligated to produce concatemers. (5) The concatemers were cloned into the vector and sequenced. Then, the occurrence of each gene tag was determined. To validate MgC-GEP, we analyzed 20 genes in *Saccharomyces cerevisiae* induced by weak acid using MgC-GEP combined with real-time reverse transcription (RT)-PCR. Compared with the results of real-time RT-PCR and the previous reports of microarray analysis, MgC-GEP can precisely determine the transcript levels of multigenes simultaneously. Importantly, MgC-GEP is a cost effective strategy that can be widely used in most laboratories without specific equipment. MgC-GEP is a potentially powerful tool for multigene expression profiling, particularly for moderate-throughput analysis.

## Introduction

With the completion of many genome projects [1], [2], [3], [4], [5], [6], [7], [8], research on addressing the roles of multiple genes in orchestrating complex cellular functions has attracted considerable attention. This requires the use of techniques that allow high-throughput analysis of such target genes. The analysis of multigene expression profiling is helpful to elucidate a series of medical and biological research questions including the dissection of basic biological processes [9], [10], the exploration of new drug targets [11], [12], [13], and the diagnosis of disease [14], [15], [16], [17]. Thus, the research of multigene expression profiling has gradually become a key point in searching specific genes or proteins.

Several methods including DNA microarray [18], massive parallel signature sequencing (MPSS) [19], serial analysis of gene expression (SAGE) [20], SuperSAGE[21], RNA-seq[22] and real-time multiplex reverse transcription (RT)-PCR [23] have been developed and widely applied in high-throughput multigene expression profiling [24], [25], [26], [27], [28], [29], [30]. However, sometimes analyzing throughput of the gene transcription profiles is moderate in number. For example, one signal transduction pathway is often composed of between 10 and 50 genes [31], [32]. A specific phenotype is often related to many gene families, which share substantial conservation at the protein and nucleotide level. Furthermore, gene expression in various samples often need be determined according to time course or different environmental stimuli. In these cases, using the methods mentioned above is not a sensible choice. For DNA microarray, though it could obtain comprehensive global expression survey, the information about some specific genes is not easily gleaned from these vast amounts of data produced [33], [34]. Moreover, some homologous genes can cross-hybridize, which makes it difficult to determine specific genes of highly homologous gene family members. For MPSS, the new tool available for conducting multigene expression profiling [29], the specific equipments necessary restrict its application widely. For SAGE and SuperSAGE, those transcripts without the *Nla*III or *Eco*P15I site may be missed which accounts for a few percent of the total transcripts in a given RNA sample [21], [35]. For RNA-seq, several informatics challenges should be considered. The efficient methods to store, retrieve and process large amounts of data must be improved to reduce errors in image analysis and base-calling and remove low-quality reads [22]. For real-time multiplex RT-PCR, it is not easy to optimize the identical reaction parameters of multiplex target amplification to obtain accurate quantification results [36], [37]. Recently, the GenomeLab™ GeXP Genetic Analysis System was developed by Beckman & Coulter (http://www.beckman.com/products/instrument/geneticanalysis/gexp_inst_dcr.asp) combined RT-PCR and capillary electrophoresis (CE). This method is more suitable for characterizing the profiling of a moderate number of genes (10–50 genes) [38], [39]. However, specific and expensive equipments are required, which also restricted its wide application. Therefore, it is necessary to establish an economical and simple method to analyze the transcript levels of dozens of genes in a particular pathway or related to a specific phenotype.

In this study, we developed a simple and reliable method, Gene Expression Profiling via Multigene Concatemers (MgC-GEP), for multigene expression profiling. We characterized the gene transcription of 20 genes in *Saccharomyces cerevisiae* that was under the weak acid stress using MgC-GEP and validated the results by real time RT-PCR. Our results show that MgC-GEP is a powerful gene expression profiling tool for a moderate number of multigenes to search and identify key players in the particular pathways of interest.

## Materials and Methods

### Strains and Vectors

*S. cerevisiae* strain W303-1A was obtained from CICIM-CU in Jiangnan University of China. The pUC19 T-vector (TaKaRa, China) was used to clone the concatemers. *Escherichia coli* strain JM109 was used for routine bacterial transformations and maintenance of plasmids.

### RNA extraction

Samples were prepared as previously described [40]. Yeast cells from overnight cultures in yeast peptone dextrose (YPD) medium were diluted in fresh YPD to an OD_600_ of 0.1 and grown at 30°C until an OD_600_ of 1–1.1 was reached. Cultures were split and then potassium sorbate (BBI, USA) was added at a final concentration of 8 mM to one half of the culture. After 20 min, both untreated and treated cultures were harvested by centrifugation at room temperature (2 min, 4000×*g*), and cells were immediately washed in ice-cold water, reharvested at 4°C, snap-frozen in liquid nitrogen and stored at −80°C. Total RNA was prepared using an SV Total RNA Isolation System (Promega, USA) according to the manufacturer's instructions. Residual DNA was digested with RNase-free DNase (DNase I, TaKaRa, China) at 37°C for 30 min. After heat inactivation for 10 min at 65°C in 2 mM EDTA, 1.5 µl total RNA solution was removed for quantification.

### Gene Expression Profiling via Multigene Concatemers (MgC-GEP)

According to the principles of GenomeLab™ GeXP Genetic Analysis System, we designed 20 pairs of multiplex gene-specific primer with a tag for the 20 genes in *S. cerevisiae* strain W303-1A and 1 pair of universal primer by using Beacon Designer 2.0 software (Bio-Rad, USA) (Table S1). Forward gene-specific primers consisted of 16–20 nucleotides corresponding to the target gene coupled to an 18-nucleotide universal forward tag sequence with a *Bam*HI site. Reverse gene-specific primers consisted of 16–20 nucleotides complementary to the target gene coupled to a 19-nucleotide universal reverse tag sequence with *Hin*dIII site. The pair of universal primers is the 18-nucleotide universal forward tag sequence and the 19-nucleotide universal reverse tag sequence. The average *T*_m_ of all 20 gene-specific primers is 67.3±1.8°C with difference from each other within 5°C. And PCR products are 85±7 bp in length. The amplification efficiencies of specific primers were examined using real-time RT-PCR (Table S3).

**Figure 1:**
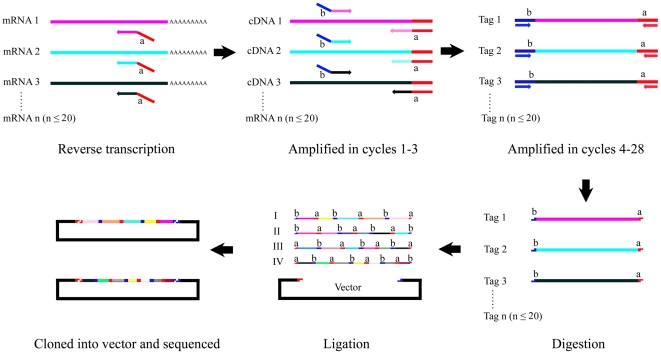
Scheme of MgC-GEP (see text for details).

For each sample, reverse transcription (RT) was followed by PCR (Fig. 1). RT reactions mixtures (25 µl) contained 500 ng RNA, 0.16 µM reverse gene-specific primer mix, 200 U reverse transcriptase (Promega, USA), 25 U RNase inhibitor (TaKaRa, China), 5 µl M-MLV 5×reaction Buffer, and 0.5 mM each dNTP. RT reactions were incubated at 70°C for 5 min, 42°C for 60 min. Subsequent PCR was done with each reaction containing 2 µl RT reaction, 0.02 µM forward primer set mix, 2.5 U Taq DNA polymerase (Bioflux, Japan), 0.2 mM each dNTP, and 10×PCR buffer (Bioflux, Japan) containing 5 mM MgCl_2_, 10 mM Tris-HCl, 50 mM KCl, 1 µM universal forward primer, and 1 µM universal reverse primer. Amplification conditions consisted of initial denaturation at 95°C for 5 min, followed by 28 cycles of 94°C for 30 s and 53°C for 30 s, 72°C for 35 s ending in a single extension cycle of 72°C for 5 min. The PCR product was extracted with PC8 (phenol∶chloroform 1∶1, pH 8.0) and precipitated with isopropanol, and dissolved in Lo TE buffer (3 mM Tris-HCl, pH 7.5; 0.2 mM EDTA, pH 7.5) after washed by 70% ethanol. The purified and concentrated product was digested by *Hin*dIII and *Bam*HI for 12 h and treated by chloroform extraction and isopropanol precipitation, and dissolved in Tris-EDTA (TE) buffer. The digested product was separated by 3% agarose gel electrophoresis. The gel containing of digested target bands was cut and centrifuged by spin column. The product was precipitated by ethanol and dissolved in Lo TE buffer. The digested products were ligated to produce concatemers by 5 U T4 DNA ligase (TaKaRa, China) and 15% PEG6000 at 16°C for 4 h. The concatemers were separated by 1.5% agarose gel electrophoresis. The region 500 bp–1200 bp was isolated and cloned in the pUC19T-vector digested by *Hin*dIII and *Bam*HI. Finally, the recombinant plasmids were transformed into *E. coli* competent cells.

We randomly selected 300 clones from each sample and inserts were sequenced using ABI PRISM 3730 automatic DNA sequence systems (SANGON Sequencing Service, Shanghai China). The sequencing primers were M13-47′ (5′-CGCCAGGGTTTTCCCAGTCACGAC-3′) and RV-M (5′-GAGCGGATAACAATTTCACACAGG-3′). The sequence and occurrence of each gene were determined and the *Act1* gene encoding Act1p, which was a ubiquitous protein involved in the filament formation, was as the internal control. The sequences were analyzed by using the local BLAST program. All samples were analyzed in triplicate.

### Real-time RT-PCR analysis

For real-time RT-PCR, the cDNA was synthesized from 1 µg of DNaseI-treated total RNA with the anchored oligo-dT primer following the manufacturer's protocol (Promega, USA). Twenty pairs of specific primers for the 20 genes in *S. cerevisiae* strain W303-1A were designed using Beacon Designer 2.0 software (Bio-Rad, USA) (Table S2). Real-time RT-PCR was performed using the SYBR-Green PCR Master Mix kit (Bio-Rad, USA) in the Light Cycler (Bio-Rad, USA). A serial dilution of the cDNA was used as standard curve to optimize the amplification efficiency with each primer pair. Three technical replicates were run for each of the standards. The cycling conditions were 95°C for 15 sec followed by 40 cycles of 95°C for 15 sec, 53.5°C for 15 sec and 72°C for 15 sec. Amplification specificity was confirmed by generating a melting curve of the PCR products. The amplified fragments were also verified by gel electrophoresis. *Act1*, which encoded Act1p, a ubiquitous protein involved in the filament formation, was amplified as the internal control. Relative target gene expression was determined with the comparative cycle threshold (C_T_) method. The ΔC_T_ value was calculated by subtracting the target C_T_ for each sample from its *Act1* C_T_ value. Every RT-PCR experiment was repeated with three biological samples and each sample was run in triplicate. The relative expression level of mRNA was analyzed between the weak acid-induced samples and non-induced samples with DPS statistical analysis software.

## Results

### Scheme of MgC-GEP

A schematic diagram of the technique is shown as Fig. 1. First, total RNA extracted from samples was reverse transcribed using 20 reverse primers each containing 19–22 nucleotides complementary to the target gene coupled with a 19-nucleotide universal reverse sequence to obtain the cDNA. The cDNA was PCR-amplified during in the initial 1–3 cycles using the specific primers to synthesize the dozens of gene-amplification products, each of which contained universal tags with specific restriction digestion sites for enzymes a and b at both termini. In this study, the universal tags included the specific restriction digestion sites of *Bam*HI and *Hin*dIII. Subsequent PCR amplifications (cycles 4–28) used universal forward and reverse primers to yield amplification products corresponding to each of the 20 specific genes. These two steps effectively condensed mRNA and reduced nonspecific amplifications. The tags corresponding to each of the 20 specific genes were digested with restriction enzyme a and b. The digested products were ligated to produce concatemers with T4 DNA ligase. The concatemers were cloned into the vector digested with restriction enzyme a and b for sequencing.

### Validation of MgC-GEP

**Figure 2:**
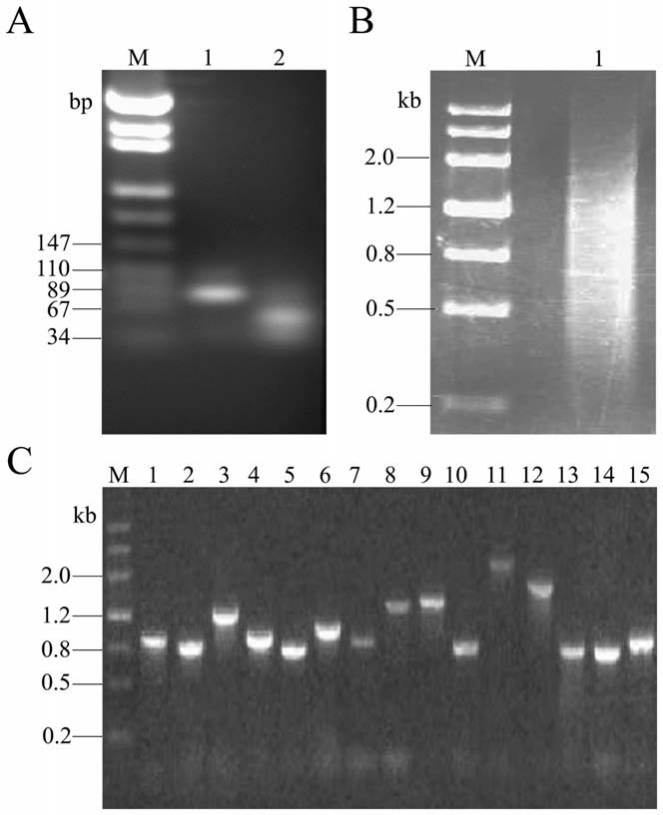
Monitoring the process of MgC-GEP. (A) Detection of the PCR products digested. Lane 1 was the PCR products without digestion. Lane 2 was the PCR products digested with *Bam*HI and *Hin*dIII. The RT-PCR products digested and undigested were separated by 3% agarose gel electrophoresis and stained with ethidium bromide. Marker (M) was the pUC18 DNA/MspI (TIANGEN). (B) The digested products were ligated to produce concatemers with T4 DNA ligase. The concatemers were analyzed by using 1.5% agarose gel and stained with ethidium bromide. Maker (M) was the MIII (DingGuo). (C) Insert size of 15 clones was estimated by PCR analysis. The PCR products were analyzed by using 1% agarose gel and stained with ethidium bromide. Lanes 1 to 15 were the different clones selected randomly. Marker (M) was the MIII (DingGuo).

To identify potentially protective genes induced by sorbate, the whole-genome transcriptional profile has been studied by whole-genome microarrays [40]. According to the results of the study, we chose to characterize the gene transcription of 20 genes including 10 up-regulated genes (*YPL122C, YNR030W, YDR343C, YGR088W, YPR149W, YCL040W, YBR054W, YNR001C, YDR533C, YDL222C*), 9 down-regulated genes (*YML123C, YEL046C, YLR180W, YLR355C, YLR419W, YLR300W, YNL300W, YLR372W, YAL059W*) and *Act1* as the internal control in *S. cerevisiae* that were under the weak acid stress to evaluate the reliability of MgC-GEP conveniently. The target genes were amplified via multiplex RT-PCR (lanes 1 in Fig. 2A) and then the RT-PCR products digested by *Bam*HI/*Hin*dIII (lanes 2 in Fig. 2A). After that, the digested 60–70-bp tags were ligated to produce concatemers and 500- to 1200-bp concatemers were collected (Fig. 2B) and cloned into the pUC19 T-vector (TaKaRa, China) digested by *Bam*HI/*Hin*dIII. Insert size of 300 clones selected randomly was estimated by PCR analysis (Fig. 2C). The size of the inserts varied from 500 to 1200 bp in length, with an average length of 800 bp. This result demonstrated that there were at least 10 tags in one clone. Inserts were sequenced using ABI PRISM 3730 automatic DNA sequence systems (Sangon, China). The sequence and occurrence of each gene were determined manually and gene expression analysis was performed using the BLAST program.

**Figure 3:**
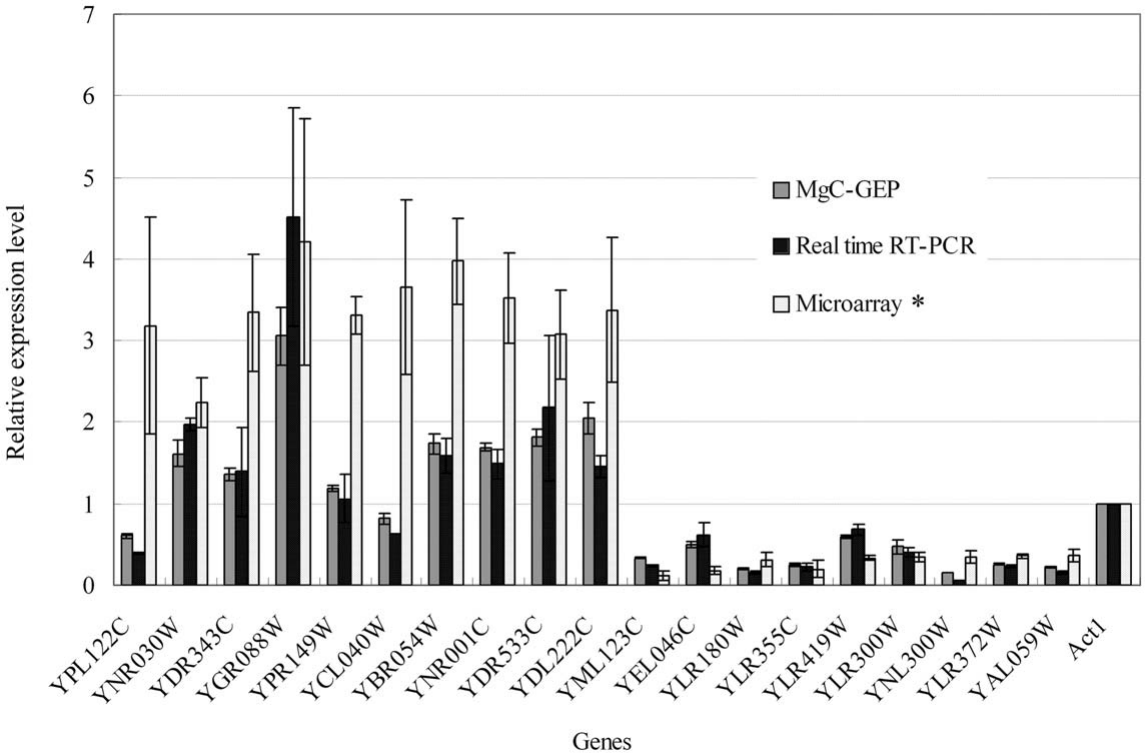
Relative expression level of 20 genes in yeast across the samples induced by weak acid and that non-induced detected by MgC-GEP, real time RT-PCR and Microarray analysis. Yeast cells of *S. cerevisiae* W303-1A strain from overnight cultures in YPD were diluted in fresh YPD to an OD_600_ of 0.1 and grown at 30°C until an OD_600_ of 1–1.1 was reached. Cultures were split and potassium sorbate (BBI) was added at a final concentration of 8 mM to one half of the culture. After 20 min, both untreated and treated cultures were harvested by centrifugation at room temperature (2 min, 4000×*g*), and cells were immediately washed in ice-cold water, reharvested at 4°C, snap-frozen in liquid nitrogen and saved at −80°C. *Microarray data was cited from previous research [40].

Using MgC-GEP, we found that the expression patterns of 16 genes in 20 selected genes were consistent with the results of previously reported microarray analysis [40]. Seven genes (*YNR030W, YDR343C, YGR088W, YBR054W, YNR001C, YDR533C, YDL222C*) were up-regulated with weak acid induction and nine genes (*YML123C, YEL046C, YLR180W, YLR355C, YLR419W, YLR300W, YNL300W, YLR372W, YAL059W*) that were repressed (Fig. 3). Notably, three genes (*YPL122C, YCL040W* and *YPR149W*) were proven to be up-regulated during weak acid induction using microarray analysis [40]. However, in MgC-GEP analysis, *YPL122C* and *YCL040W* were down-regulated and the transcription level of *YPR149W* did not noticeably change during weak acid induction. To validate MgC-GEP, we also detected the transcription of the 20 selected genes using real time RT-PCR. The results showed that the expression patterns of 20 genes analyzed by real time RT-PCR were consistent with the results of MgC-GEP analysis. These results demonstrated that MgC-GEP can accurately determine the transcript levels of at least 20 genes at once.

## Discussion

In this study, we present a novel method for multigene expression profiling, designated as MgC-GEP. To validate MgC-GEP, we identified the transcript levels of 20 genes stimulated with weak acid in *S. cerevisiae* using the MgC-GEP and real-time RT-PCR, respectively. Additionally, we have successfully detected the transcription of 8 genes of Toll pathway of *Locusta migratoria manilensis* in eight different tissues at six time points using MgC-GEP (unpublished data). It demonstrated that MgC-GEP could exactly determine the transcript levels of a moderate number of genes at once effectively.

In MgC-GEP, two initial steps were performed to synthesize cDNA using specific primers containing universal tags with specific digestion sites at both termini. The universal tags, containing the specific digestion sites, will facilitate the formation of concatermers. It offers another advantage, namely, the simplification of the subsequent step by making it possible to analyze the fragments by polyacrylamide gel electrophoresis or agarose gel electrophoresis. Importantly, MgC-GEP is based on sequencing technique and can be freely applied in most laboratories without the acquisition of specific equipments.

Multiple primer design is a very important step in MgC-GEP. First of all, the calculated primer melting temperature (Tm) and the size of PCR products should be similar so that all primers anneal at same temperatures during the PCR temperature cycling for better balance between PCR products in a multiplex reaction [41]. Moreover, high quality primers are essential for successful multiplex amplification reactions. During 3–28 cycles in the amplification, the universal primers are more competitive than the chimeric primers due to their higher abundance. At this time, different genes are amplified with the same universal primer pairs. Therefore, the chimeric primers and universal primers could lead to the same efficiency for each amplicon.

When choosing a method for quantifying gene expression, there are several basic considerations, including specificity, throughput, sensitivity, cost and data analysis. Like SAGE and SuperSAGE, MgC-GEP is based on the sequential analysis of short cDNA sequence tags [20], [21]. Each tag is derived from a defined position within a transcript. For specificity, the size (>60 bp) of the tags is long enough to be identified as the corresponding gene and the frequency of each tag numbered provides an accurate measurement of its expression level. Closely related gene sequences could be discriminated through precise primer designing and sequencing in MgC-GEP. Specifically, this method could allow the accurate determination of the expression levels of multiple transcripts that could lead to the false positive of an expression differential by hybridization due to the high identity at nucleotide level. For sensitivity, it is possible to detect poorly expressed genes with high sensitivity using MgC-GEP since it is a PCR-based technique. For throughput, the transcript levels of at least 20 genes could be monitored at once using MgC-GEP. It was more practical to monitor the transcript levels of 10–50 genes under the various conditions or at different times than the real-time RT-PCR, DNA microarray and SAGE. For real-time multiplex RT-PCR, if one template is much more abundant than the other templates in the same reaction tube, the reaction components will be depleted before the lower-abundance targets have amplified sufficiently to be detected [23]. For DNA microarray and SAGE, though thousands of genes could be determined, the large cost of multiple samples may be the main constraint [18]. For example, detecting the expression profiling of 10–50 genes in kinetics studies or comparing the effects of a large number of drugs is costly. The GeXP method is a good choice for multigene expression profiling of multiple samples, but the necessary equipments such as a fluorescence scanner (detector) and a capillary electrophoresis machine are required to generate the data [38], [39]. This is inconvenient for laboratories that do not possess this equipment. MgC-GEP can be applied in most laboratories and done readily without additional cost and equipment. Another advantage is in data analysis, MgC-GEP can use Blast analysis to detect and count tags from sequence files, thus can analyze the experimental data without the necessity of professional software. Although there are distinct advantages to MgC-GEP, there are also two limitations: MgC-GEP does not determine the absolute expression level of transcripts but the relative abundance of selected transcripts, and the sensitivity of MgC-GEP depends on number of clones sequenced, thus detection of rare transcripts would sequence more clones which will increase the cost.

In conclusion, MgC-GEP adopts covnentional RT-PCR, restriction enzyme digestion, concatemer ligation, bacterial transformation and DNA sequencing to assess mRNA profiles without using high-throughput expensive methodologies such as microarray hybridization or deep sequencing. No specific equipment is required in this method. This strategy could be of great benefit for labs with limited funds or limited access to microarray or next-generation sequencing facilities. Thus, MgC-GEP should be a potentially powerful tool for multigene expression profiling in different tissues, during development, or during specific pathologies in both basic and pharmaceutical research.

## Supporting Information

Table S1 **Multiplex gene-specific primers and universal primers.** *Underlined sequences are restriction sites. Italic letters are the universal sequences.
(DOC)

Table S2 **Primer sequence for real-time RT-PCR.**
(DOC)

Table S3 **The amplification efficiencies of specific primers.** The amplification efficiencies of specific primers were determined using real-time RT-PCR described in Materials and Methods.
(DOC)
