# Supplementary material for: Gene Expression Profiling via Multigene Concatemers
Source: PLoS One. 2011 Jan 18;6(1):e15711. doi: 10.1371/journal.pone.0015711 (PMC3022625; doi:10.1371/journal.pone.0015711)
Supplement: Table S1 — Multiplex gene-specific primers and universal primers. *Underlined sequences are restriction sites. Italic letters are the universal sequences. (DOC) [file pone.0015711.s001.doc]

**SUPPLEMENTARY TABLES**

Table S1. Multiplex gene-specific primers and universal primers

| Gene name | Forward primer (5'-3') * | Reverse primer (5'-3') * | PCR product (bp) | Digested fragment (bp) |
| --- | --- | --- | --- | --- |
| *YPL122C* | *AGGTGACAGGATCCAATA*CGGTGTGGTTGTAGAGG | *GTACGACTCAAAGCTTGGA*CCAACAAGTCCAGGCTAA | 81 | 61 |
| *YNR030W* | *AGGTGACAGGATCCAATA*TTCGGTATCTTTGGCTTG | *GTACGACTCAAAGCTTGGA*TGATACTGATGGCGGAAC | 80 | 60 |
| *YDR343C* | *AGGTGACAGGATCCAATA*GAGGTGCCAACTACGACG | *GTACGACTCAAAGCTTGGA*TATCATCGTGAGCCATTTC | 78 | 58 |
| *YGR088W* | *AGGTGACAGGATCCAATA*CGGATACTGGTTTTGAA | *GTACGACTCAAAGCTTGGA*TTCAGCAGCCTTATCTCC | 81 | 61 |
| *YPR149W* | *AGGTGACAGGATCCAATA*ACCCTGATGTCTGTTTTC | *GTACGACTCAAAGCTTGGA*CAGAACCAAAGGCACCA | 86 | 66 |
| *YCL040W* | *AGGTGACAGGATCCAATA*ACCTTTCCAGTTGTCATCC | *GTACGACTCAAAGCTTGGA*GTCAATTTCAATATGCGACA | 83 | 63 |
| *YBR054W* | *AGGTGACAGGATCCAATA*AAATTTTTTACGCTAGATACG | *GTACGACTCAAAGCTTGGA*TGGGAAAGCCAAAAACC | 81 | 61 |
| *YNR001C* | *AGGTGACAGGATCCAATA*ACTGAGGCTTCGTTCTACAC | *GTACGACTCAAAGCTTGGA*TAGCTCTGGCAACACCA | 83 | 63 |
| *YDR533C* | *AGGTGACAGGATCCAATA*ACTGGTAACAGGTGTGAATC | *GTACGACTCAAAGCTTGGA*TCTTACGGCAGTGGAGTG | 87 | 67 |
| *YDL222C* | *AGGTGACAGGATCCAATA*AGCCTCAACCTACAACCAC | *GTACGACTCAAAGCTTGGA*GAAAGAACTTGCCATTGC | 88 | 68 |
| *YML123C* | *AGGTGACAGGATCCAATA*CCGCACAAGAACAAGATGG | *GTACGACTCAAAGCTTGGA*TCTTCATCACTGGTGTCG | 89 | 69 |
| *YEL046C* | *AGGTGACAGGATCCAATA*TGTCTCAAGCGATGGTGG | *GTACGACTCAAAGCTTGGA*TAGTCACCGTTGGAAGGAA | 83 | 63 |
| *YLR180W* | *AGGTGACAGGATCCAATA*AGAGCGCAACTAAAGTCCG | *GTACGACTCAAAGCTTGGA*TGTCTCTTGGGATGACTTTT | 87 | 67 |
| *YLR355C* | *AGGTGACAGGATCCAATA*CGATGCCGCTCAATCAGAA | *GTACGACTCAAAGCTTGGA*CCTTGGTCAACAATGGCT | 90 | 70 |
| *YLR419W* | *AGGTGACAGGATCCAATA*CTCGGCTATTGGGCTTGC | *GTACGACTCAAAGCTTGGA*CCCACAAGTTACACAGCGT | 92 | 72 |
| *YLR300W* | *AGGTGACAGGATCCAATA*TATGACCACGGTTCCCTCG | *GTACGACTCAAAGCTTGGA*CCACCAATGTTGACACCAC | 87 | 67 |
| *YNL300W* | *AGGTGACAGGATCCAATA*GTAGATGTTACCACCACCCC | *GTACGACTCAAAGCTTGGA*TGGAGACCATAGATGTAGTA | 83 | 63 |
| *YLR372W* | *AGGTGACAGGATCCAATA*ACTGGTGTCAAGACCTCTA | *GTACGACTCAAAGCTTGGA*GCTTTCCTGGAAGAGACCT | 81 | 61 |
| *YAL059W* | *AGGTGACAGGATCCAATA*CCATTTCTCGTGCCAAGTAC | *GTACGACTCAAAGCTTGGA*TATCCCAGCCAGCCTTTC | 88 | 68 |
| *Act1* | *AGGTGACAGGATCCAATA*GTGGTTACTCTTTCTCCAC | *GTACGACTCAAAGCTTGGA*CGGACAATTTCTCTTTCAG | 80 | 60 |
| *Universal*  *Primer* | *AGGTGACAGGATCCAATA* | *GTACGACTCAAAGCTTGGA* |  |  |

*Underlined sequences are restriction sites. Italic letters are the universal sequences.
