# Supplementary material for: Gene Expression Profiling via Multigene Concatemers
Source: PLoS One. 2011 Jan 18;6(1):e15711. doi: 10.1371/journal.pone.0015711 (PMC3022625; doi:10.1371/journal.pone.0015711)
Supplement: Table S2 — Primer sequence for real-time RT-PCR. (DOC) [file pone.0015711.s002.doc]

**SUPPLEMENTARY TABLES**

Table S2. Primer sequence for real-time RT-PCR

| Gene name | Forward primer (5'-3') | Reverse primer (5'-3') | PCR product size (bp) |
| --- | --- | --- | --- |
| *YPL122C* | CGCATTGGAAGTTGGCAAAG | TCGGAAGTCAGCATCAAAGC | 156 |
| *YNR030W* | CAGGAGCAGCACATCTATGG | CTCGCCGCCTGGATAATTC | 149 |
| *YDR343C* | TCATGGGCTGTTTGGTCTTC | GTCGTAGTTGGCACCTCTTC | 161 |
| *YGR088W* | CCACGTCTTGTCGGATACTG | GTTCGGGTGTCATTGTTTGC | 161 |
| *YPR149W* | AACACCCAACATAGGCACAG | TGATACCAACGGCCAACAC | 196 |
| *YCL040W* | TCAGACTGCCCACCACTC | CACAACGGAACCATCACAAC | 176 |
| *YBR054W* | AGGAGCACCCAGGTTACAG | AGCAATCAACCAGCAGACAG | 167 |
| *YNR001C* | TGGTCGTGCCAATCAAGAAG | AACTCTCCCTGCGTTCAAAG | 121 |
| *YDR533C* | TGGGATGAGCATTCCTTAGC | TCGGCATTCACCTCTTTTGG | 125 |
| *YDL222C* | ACAGGTTGCTATGTGAAGGC | TGGTCCGAGTAGGTAGAGTG | 173 |
| *YML123C* | GCTGGTGTTGGTTTCTTGAC | TTGACTTGGACCTGGCATAC | 108 |
| *YEL046C* | TGTCCAACCAGATTGCCATC | GGAACCACCATCGCTTGAG | 130 |
| *YLR180W* | TCGTCATCGGTGGTCCTC | CGGCATAAGCGGCAGAAC | 144 |
| *YLR355C* | TTACGGTTCCCAAGGTTACG | GAGCGGCATCGGACAAC | 197 |
| *YLR419W* | CAACTGCCTGCCTGGAAG | CGACCTGAGTGGATTTACCC | 106 |
| *YLR300W* | GTTTGCTGCCGCTTTGAC | TGCCTCCACATAACGTCTTG | 184 |
| *YNL300W* | ACCGTTGCTGCCATTGC | GGTGGTGGTGTTAGCTTGG | 195 |
| *YLR372W* | CAGTTGCCGACCAGTTCC | ACCCGTTAGCCAAGAAAGTC | 189 |
| *YAL059W* | ATCGGATGCTCTTGAACCAG | CTCATTCTTGGCTGCTGTTC | 103 |
| *Act1* | TCTGAGGTTGCTGCTTTGG | CCGACGATAGATGGGAAGAC | 101 |
