# Supplementary material for: Gene Expression Profiling via Multigene Concatemers
Source: PLoS One. 2011 Jan 18;6(1):e15711. doi: 10.1371/journal.pone.0015711 (PMC3022625; doi:10.1371/journal.pone.0015711)
Supplement: Table S3 — The amplification efficiencies of specific primers. The amplification efficiencies of specific primers were determined using real-time RT-PCR described in Materials and Methods. (DOC) [file pone.0015711.s003.doc]

**SUPPLEMENTARY TABLES**

Table S3. The amplification efficiencies of specific primers

| Gene | Amplification efficiency (%) |
| --- | --- |
| *YPL122C* | 100.1 |
| *YNR030W* | 99.0 |
| *YDR343C* | 98.9 |
| *YGR088W* | 96.6 |
| *YPR149W* | 96.6 |
| *YCL040W* | 97.6 |
| *YBR054W* | 96.0 |
| *YNR001C* | 98.4 |
| *YDR533C* | 99.3 |
| *YDL222C* | 96.1 |
| *YML123C* | 104.5 |
| *YEL046C* | 97.2 |
| *YLR180W* | 98.2 |
| *YLR355C* | 98.6 |
| *YLR419W* | 97.0 |
| *YLR300W* | 103.8 |
| *YNL300W* | 99.9 |
| *YLR372W* | 97.6 |
| *YAL059W* | 95.6 |
| *Act1* | 98.7 |

The amplification efficiencies of specific primers were determined using real-time RT-PCR described in MATERIALS AND METHODS.
